# Chemical Composition of Cuticle and Barrier Properties to Transpiration in the Fruit of *Clausena lansium* (Lour.) Skeels

**DOI:** 10.3389/fpls.2022.840061

**Published:** 2022-05-12

**Authors:** Hua Huang, Ling Wang, Diyang Qiu, Yusheng Lu

**Affiliations:** ^1^Institute of Fruit Tree Research, Guangdong Academy of Agricultural Sciences; Key Laboratory of South Subtropical Fruit Biology and Genetic Resource Utilization, Ministry of Agriculture and Rural Affairs, Guangdong Provincial Key Laboratory of Tropical and Subtropical Fruit Tree Research, Guangzhou, China; ^2^Sericultural & Agri-Food Research Institute Guangdong Academy of Agricultural Sciences, Key Laboratory of Functional Foods, Ministry of Agriculture and Rural Affairs, Key Laboratory of Agricultural Products Processing, Guangzhou, China

**Keywords:** wampee fruit, cuticular waxes, cutin monomers, transpiration, barrier properties

## Abstract

The plant cuticle, as a lipid membrane covering aerial plant surfaces, functions primarily against uncontrolled water loss. Herein, the cuticle chemical composition and the transpiration of wampee fruit (*Clausena lansium* (Lour.) Skeels) at the green, turning, and yellow stages in cultivars of “Jixin” and “Tianhuangpi” were comprehensively studied. The coverage of wax and cutin monomers per unit of fruit surface area at the green stage was lower in “Jixin” than in “Tianhuangpi” and increased gradually during development. Cutin monomers accumulated ranging from 22.5 μg cm^−2^ (green) to 52.5 μg cm^−2^ (turning) in “Jixin” and from 36.5 μg cm^−2^ (green) to 81.7 μg cm^−2^ (yellow) in “Tianhuangpi.” The total composition of waxes ranged between 6.0 μg cm^−2^ (green) and 11.1 μg cm^−2^ (turning) in “Jixin,” while they increased from 7.4 μg cm^−2^ (green) to 16.7 μg cm^−2^ (yellow) in “Tianhuangpi.” Cutin monomers were dominated by ω-, mid-dihydroxy fatty acids (over 40%), followed by multiple monomers of α,ω-dicarboxylic acids with or without added groups, α-monocarboxylic acids with or without ω- or mid-chain hydroxy or mid-epoxy groups, primary alcohols, and phenolics. The very-long-chain (VLC) aliphatic pattern of cuticular waxes was prominently composed of *n*-alkanes (ranging from 21.4% to 39.3% of total wax content), fatty acids, primary alcohols, and aldehydes. The cyclic waxes were dominated by triterpenoids (between 23.9 and 51.2%), sterols, and phenolics. Water loss in wampee fruit exhibited linear changes over time, indicating an overall monofunctional barrier to transpiration. Permeance for water in wampee fruit was higher at the green stage than at the yellow stage in both “Jixin” and “Tianhuangpi,” which showed a negative correlation with the changes of VLC *n*-alkanes. The results showed the cuticular chemicals, including cutin monomers and waxes, in wampee fruit and further indicated the potential contributions of the cuticular chemical composition to the physiological functions in fruits.

## Introduction

*Clausena lansium* (Lour.) Skeels, named wampee or “huangpi (黄皮)” in Chinese for the yellow skin in ripe fruits, is a typical subtropical to tropical fruit. Wampee is native to south China and is also planted in other regions of southern Asia (Rodrigues et al., 2018). Almost all parts of the wampee, including leaves, seeds, roots, and fruits, contain abundant carbazole alkaloids, triterpenoids, and amides, exhibiting strong antioxidant, anticancer, and anti-inflammatory activities (Prasad et al., 2010; Shen et al., 2017; Peng et al., 2019). Therefore, wampee is potentially an economically important plant due to its nutritional and medicinal value.

The plant cuticle is a continuous lipid membrane covering all the aerial plant organs against uncontrolled water loss, pathogen infection, and cracking (Yeats and Rose, 2013). This extracellular membrane has been well studied and is composed of soluble waxes, containing very-long-chain aliphatics and cyclic triterpenoids; the cutin matrix is constructed by C_16_- and C_18_-type fatty acid derivatives as well as a small content of phenolics (Jetter et al., 2008; Fich et al., 2016). It has long been established that the barrier to nonstomatal transpiration in plants is largely provided by the cuticle, especially the wax pattern (Riederer and Schreiber, 2001). In addition, both cutin polymers and waxes provide a mechanical barrier to pathogen invasion or as signal components involved in the germination of conidia (Hansjakob et al., 2010; Serrano et al., 2014). Though the phytochemicals of various organs in wampee have been widely investigated, the chemical compositions of the outermost cuticle have not yet been addressed.

Wampee belongs to the Rutaceae family being a distant relative of citrus, and the size of the fruit resembles grape berry and cherry fruit (Rodrigues et al., 2018). Unlike citrus, in which the peel tissue of fruits is independent of the edible parts, the peel adheres together with the pulp in wampee fruit. Wampee is a perishable fruit, which is susceptible to mechanical damages, browning, and disease infection after harvest (Shao et al., 2020). Meanwhile, pathogen invasion as a factor-causing fruit softening and cracking has been a concern in wampee fruit production (Zhou et al., 2015). It has been widely reported that the chemical composition and the structural arrangements of the cell wall or cuticle, as well as the fruit surface properties, are pivotal in maintaining the fruit quality (Ríos et al., 2015; Winkler et al., 2020). Accordingly, the peel tissues may play important roles in affecting fruit quality changes during development and postharvest storage.

In addition, the most popular product of wampee is dried fruit. Therefore, the desiccation process is important for product quality. The tolerance to desiccation in seeds increases during development (Fu et al., 2008), and the drying conditions are also important for the quality of the final product (Chokeprasert et al., 2005). Therefore, elucidating the characteristics of water loss and its barrier property to transpiration might be helpful in improving the production of dry wampee. However, the transpiration property and the potential barrier property of the hydrophobic cuticle in wampee fruit are still unclear.

The goal of this study was to characterize the chemical composition of cuticles and the potential effect of various cuticular components on the transpiration barrier properties in wampee fruit. Previous studies on the varieties of wampee fruit have found that the sour wampee fruit with a common dark yellow surface contained higher antioxidants and a higher antioxidant activity than the sweet wampee fruit with a light yellow surface (Ye et al., 2019). In this study, “Jixin,” the common sour wampee fruit, and “Tianhuangpi,” with high sugar content and sweet flavor, were selected to be comparatively studied. Changes in cuticle composition, including waxes and cutin monomers, as well as the transpiration at the green, turning, and yellow ripe stages, were studied in detail. The chemical composition of the cuticle and the potential contributions to the transpiration barrier properties in wampee fruit are also comprehensively discussed.

## Materials and Methods

### Plant Materials and Reagents

Wampee fruits (*Clausena lansium* (Lour.) Skeels) at the green (about 60 days after anthesis, DAA), turning (about 75 DAA), and yellow ripe (about 90 DAA) stages in cultivars of “Jixin” and “Tianhuangpi” were harvested from an orchard in Guangzhou, P. R. China (23°30'N, 113°30'E). The fruits were transported back to the laboratory immediately, and the fruits with uniformity of shape, color, and size were selected for further experiments. At each developmental stage, 15 individual fruits were selected to determine the transpiration, and at least 10 fruits were used to isolate the cuticular membranes for further chemical analysis. All the experimental reagents used were in analytical grade and were prepared following the methods given in the previous reports (Huang and Jiang, 2019).

### Preparation of Cuticular Membranes From Wampee Fruit

Cuticular membranes from wampee fruit at different stages were prepared following the previous methods with minor modifications (Huang and Jiang, 2019). The fruits with uniform shape and size at each stage were randomly selected to isolate cuticular membranes. Because of different fruit sizes during development, peel disks from the middle position of wampee fruit at the green, turning, and yellow stages were prepared using a puncher with diameters of 0.5, 0.8, and 1.0 cm, respectively. The cuticular membranes were isolated by immersing the peel disks in 10 mM citric acid buffer containing 1% (w/v) pectinase and 1% (w/v) cellulase (Beijing Solarbio Science & Technology Co., Ltd., Beijing, China) and incubated under 37°C for 2–3 days. Once most of the tissues were removed from the fruit skin, the cuticular membranes were then washed by 10 mM sodium tetraborate decahydrate (Solarbio Science and Technology Co., Ltd., Beijing, China) to absorb the contamination of free fatty acids. The cuticular membranes were further rinsed with distilled water and air-dried for further analysis.

### Cuticular Wax and Cutin Monomer Extraction

Cuticular waxes and cutin monomers were extracted in series from isolated cuticular membranes according to the previous methods with minor modifications (Huang et al., 2017). The isolated cuticular membranes were completely dipped in chloroform (Guangzhou Chemical Reagent Factory, China) with a mild temperature of around 40°C to better release the soluble waxes. The extraction time was set for 2 min. Each sample was extracted three times and combined with the extracts. Then, *n*-tetracosane (Sigma–Aldrich, Shanghai, China) was added as an internal standard to evaluate the cuticular contents. The solvent in the extracts was evaporated by gentle nitrogen gas until they dried for further analysis. After that, the membranes that have been removed of soluble waxes were subsequently depolymerized in boron trifluoride with methanol (BF_3_-methanol, 10%, ~1.3 M, Sigma–Aldrich, Shanghai, China) and incubated for 16 h at 70°C. After membranes were lysed, *n*-dotriacontane (Sigma–Aldrich, Shanghai, China) as an internal standard was added. Saturated aqueous sodium chloride solution and chloroform were added in series to extract the cutin monomers. The organic phase was collected and evaporated to dryness under a gentle stream of nitrogen gas for further analysis.

### Gas Chromatography–Mass Spectrometry

To detect the chemical composition of cuticular waxes and cutin monomers, the above-prepared extracts were derivatized with pyridine (Shanghai Aladdin Bio-Chem Technology Co., Ltd., Shanghai, China) and *N,O*-bis (trimethylsilyl) trifluoroacetamide (BSTFA, Sigma–Aldrich, Shanghai, China) for 30 min at 70°C. The chemical components were analyzed using a temperature-controlled capillary gas chromatography (7890B GC System; Agilent Technologies, USA) instrument equipped with a mass spectrometric detector (*m*/*z* 50–750, MSD 5977B; Agilent Technologies, USA). Single compounds were identified based on their electron ionization mass spectra using authentic standards, the mass spectral library of Wiley 10^th^ (John Wiley & Sons), NIST 2014 (W10N14), or by interpretation of the spectra information, including the retention times and fragments of m/z, which were analyzed by comparison with data from the literature (Holloway, 1982; Jetter et al., 2008) or online database (https://www.lipidmaps.org/resources/lipidweb/lipidweb_html/index.html).

To quantify wax and cutin monomer components, the extracts were analyzed using a capillary gas chromatography instrument equipped with a flame ionization detector (7890A, GC System; Agilent Technologies, Santa Clara, CA, USA) and on-column injection with a capillary column (30 m × 0.32 mm, DB-1 ms, 0.1 μm film; J&W Scientific, Agilent Technologies, USA). To separate cuticular wax compounds, 1-μL samples were injected at 50°C; after 2 min at 50°C, the temperature was raised to 200°C at 40°C min^−1^ and held for 2 min and then raised to 320°C at 3°C min^−1^ and held for 30 min. For separation of the cutin monomers, 1-μL samples were injected at 50°C; after 1 min at 50°C, the temperature was raised to 150°C at 10°C min^−1^ and held for 2 min and then raised to 320°C at 3°C min^−1^ and held for 30 min. The area under the peaks was compared with that of the internal standards to obtain the quantity of cuticular waxes and cutin monomers. Five repetitions for each cultivar at each stage were performed for both wax and cutin analysis. The average chain length of the VLC acyclic compounds was calculated following the methods reported by Huang et al. (2017).

### Determination of Fruit Transpiration

The transpiration of the fruit at different stages was determined gravimetrically by recording the weight loss with the extension of time (Huang et al., 2017). In brief, an intact fruit without any defects in a total of 12–15 samples from each developmental stage and cultivar was carefully selected. Before measurement, fruit samples were saturated with water by dipping fruit stalks in water overnight. Then, the fruit pedicel scars were sealed with paraffin wax to avoid stalk water loss. The weight loss of fruit vs. time was recorded every 2 h over six times and extended to over 24 h, and the atmosphere temperature was controlled at 25°C. The measurement was taken using an analytic electronic balance with a precision of 0.1 mg (BSA-224S, Sartorius, Beijing, China). The dynamic changes in accumulative transpiration (flux of water vapor, *F* in g m^−2^) per unit of fruit surface area (A, m^2^) vs. time (Δ*t*) were analyzed. The fruit surface area was obtained by regarding the fruit as an ellipsoid or a sphere shape. Subsequently, permeance for water (*P* in m s^−1^) of wampee fruit was obtained according to the transpiration rate *via* a driving force:


$$\begin{array}{l} P=\frac{F}{A^{*}C_{w}^{*}\left(a_{fruit}-a_{air}\right)} \end{array}$$

The concentration of water vapor under the saturated status (C_w_) was referred to from the tabulated values (Nobel, 2009). The fruit water activity (a_fruit_) was assumed as a unit. The air water activity (a_air_) over silica gel was regarded to be close to zero.

### Statistical Analysis

Statistical analyses of all the experimental data were performed by SPSS (23, IBM Corp., Armonk, NY, USA) and SigmaPlot 12.5 (Systat Software, Inc., San Jose, CA, USA). Comparison analyses were carried out by one-way analysis of variance, and the differences between the two groups were analyzed at a level of 0.05. All the graphs were performed by SigmaPlot 12.5.

## Results

### Changes in Fruit Surface Area at Different Developmental Stages

**Figure 1:**
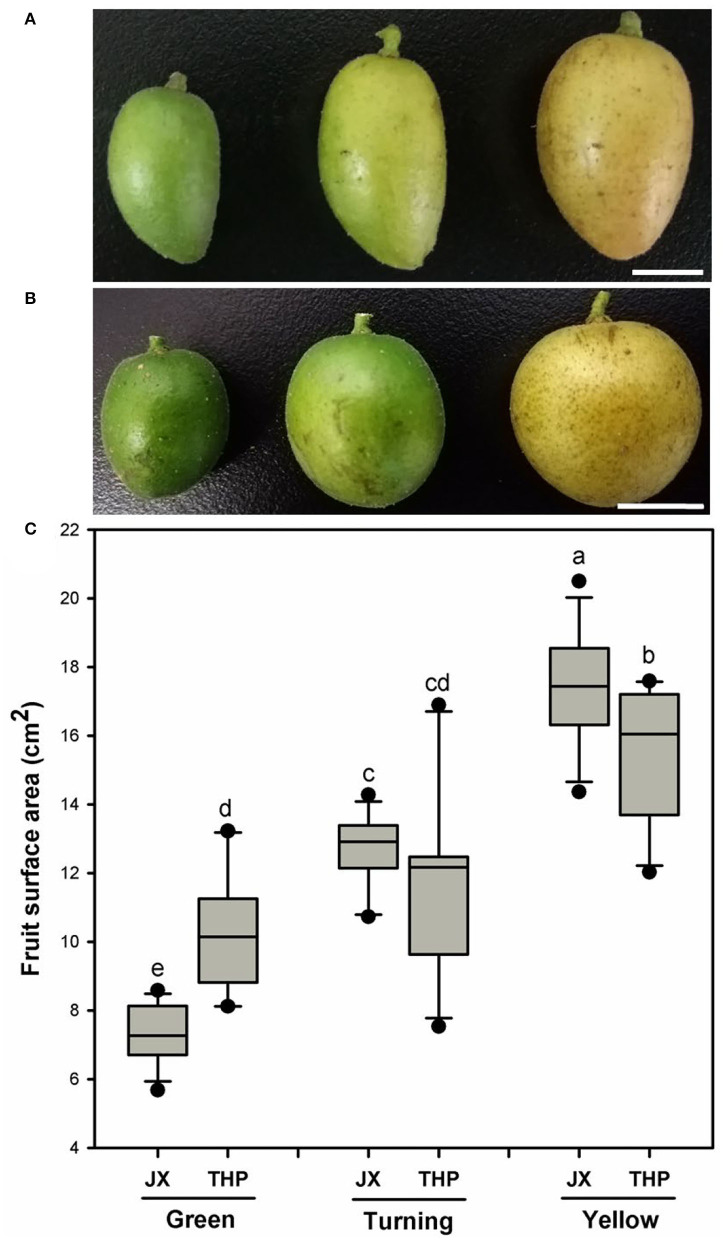
Changes in fruit appearance and surface area changes at different developmental stages for wampee (*Clausena lansium* (Lour.) Skeels). The overall changes of “Jixin” —JX **(A)**, and “Tianhuangpi” —THP **(B)**, as well as the fruit surface areas **(C)** at the green, turning, and yellow ripe stages. Data are presented as the mean ± standard deviation (*n* = 12). Scale bars in **(A)** and **(B)** are 1 cm. Different letters indicate the significant differences at the level of 0.05.

The fruit shape differed among the variety of cultivars and had different developmental stages. The fruit resembled an ellipsoid at all the developmental stages in “Jixin” (Figure 1A). In contrast, it was close to an ellipsoid shape at the green stage and shifted to a more sphere-like shape at the turning and yellow stages in “Tianhuangpi” (Figure 1B). Fruit size, measured as the surface area, increased gradually with the development and was calculated on the basis of the assumed shape of the fruit. The fruit surface area was the lowest at the green stage and increased rapidly by 70% from 7.3 cm^2^ (green) to 12.7 cm^2^ (turning) and to 17.3 cm^2^ in the yellow fruit (2.5-fold of the green fruit) in “Jixin.” It increased slowly by 15% from 10.3 cm^2^ (green) to 11.8 cm^2^ (turning) and by 35% in the yellow fruit (13.7 cm^2^) in “Tianhuangpi” (Figure 1C).

### Cuticular Chemicals Detected in Wampee Fruit

**Figure 2:**
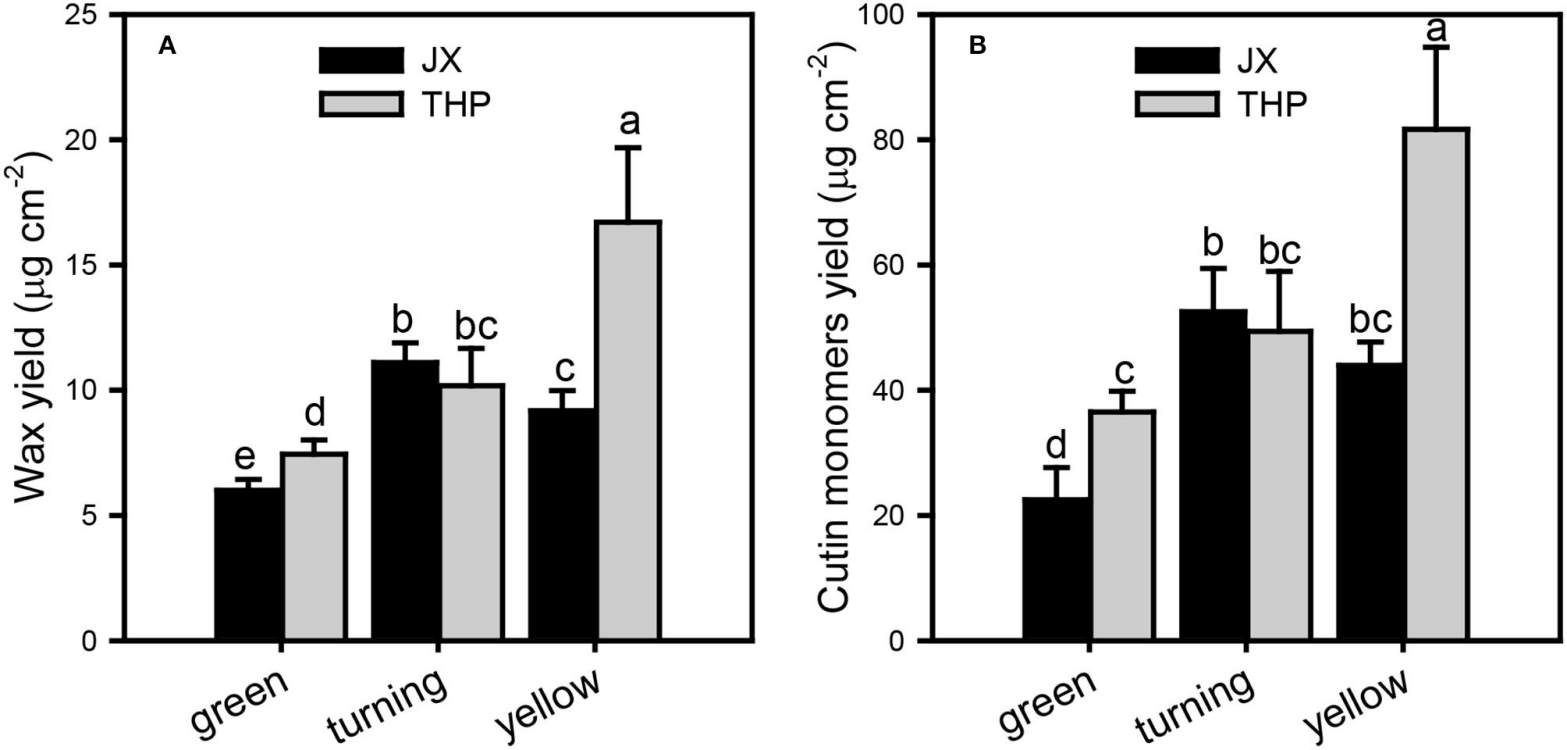
The overall cuticular chemical composition in wampee fruit (*Clausena lansium* (Lour.) Skeels). Total cuticular waxes **(A)** and total cutin monomers **(B)** in the three developmental stages of “Jixin” —JX and “Tianhuangpi” —THP. Data are presented as the mean ± standard deviation (*n* = 5). Different letters indicate the significant differences at the level of 0.05.

The overall accumulation of waxes and cutin monomers per unit of surface area was lower in “Jixin” than in “Tianhuangpi.” The contents of cuticle chemicals were lowest at the green stage, increased until the turning stage, and decreased thereafter in “Jixin.” In contrast, they accumulated gradually during the whole development of “Tianhuangpi” (Figure 2). Cuticular wax was covered between 6.0 μg cm^−2^ (green) and 11.1 μg cm^−2^ (turning) in “Jixin” and ranged from 7.4 μg cm^−2^ (green) to 16.7 μg cm^−2^ (yellow) in “Tianhuangpi” (Figure 2A). Cutin monomers accumulated from 22.5 μg cm^−2^ at the green stage to 52.5 μg cm^−2^ at the turning stage in “Jixin” and varied from 36.5 μg cm^−2^ at the green stage to 81.7 μg cm^−2^ at the yellow stage in “Tianhuangpi” (Figure 2B). As a result, the ratio of total wax over cutin monomers was maintained at around 0.21 (Table 1).

**Table 1:** Overall cuticular chemicals including waxes and cutin monomers of wampee fruit (*Clausena lansium* (Lour.) Skeels) in two cultivars of “Jixin” and “Tianhuangpi”.

	**“Jixin”**			**“Tianhuangpi”**			**Unit**
	**Green**	**Turning**	**Yellow**	**Green**	**Turning**	**Yellow**	
Aliphatics	1.91 ± 0.49^c^	4.21 ± 0.68^b^	3.97 ± 0.59^b^	1.97 ± 0.29^c^	4.50 ± 1.68^abc^	8.27 ± 2.10^a^	μg cm^−2^
Cyclics	2.64 ± 0.37^c^	4.48 ± 0.83^ab^	3.73 ± 0.25^b^	4.07 ± 0.64^b^	3.88 ± 0.35^b^	5.41 ± 0.45^a^	μg cm^−2^
Aliphatics/cyclics	0.73 ± 0.22^bc^	0.97 ± 0.28^ab^	1.07 ± 0.23^ab^	0.50 ± 0.15^c^	1.19 ± 0.52^ab^	1.52 ± 0.29^a^	ratio
ACL	28.01 ± 0.81^ab^	28.76 ± 0.21^b^	29.29 ± 0.01^a^	28.46 ± 0.28^b^	29.22 ± 0.13^a^	29.08 ± 0.13^a^	carbons
C16/C18 monomers	9.14 ± 1.68^ab^	7.15 ± 1.11^b^	6.49 ± 0.68^b^	10.83 ± 1.02^a^	7.58 ± 0.82^b^	7.44 ± 1.56^b^	ratio
Wax/cutin	0.28 ± 0.07^a^	0.21 ± 0.03^a^	0.21 ± 0.01^a^	0.20 ± 0.01^a^	0.21 ± 0.07^a^	0.21 ± 0.05^a^	ratio
Cuticle yield	32.81 ± 6.04^d^	71.71 ± 8.08^b^	59.66 ± 4.47^bc^	50.91 ± 4.73^c^	66.30 ± 8.89^bc^	109.21 ± 16.60^a^	μg cm^−2^

*The cuticle yield, coverage of VLC aliphatics, cyclics (μg cm^−2^), the ratio of total wax vs. cutin monomers, aliphatics vs. cyclics, and C_16_- vs. C_18_-type monomers in cutin, and the weight average chain length (ACL) of aliphatics in waxes*.

*Data are given as mean values with standard deviation (n = 5)*.

*Different letters indicate the significant differences at the level of 0.05*.

### Accumulation of Cutin Monomers

**Figure 3:**
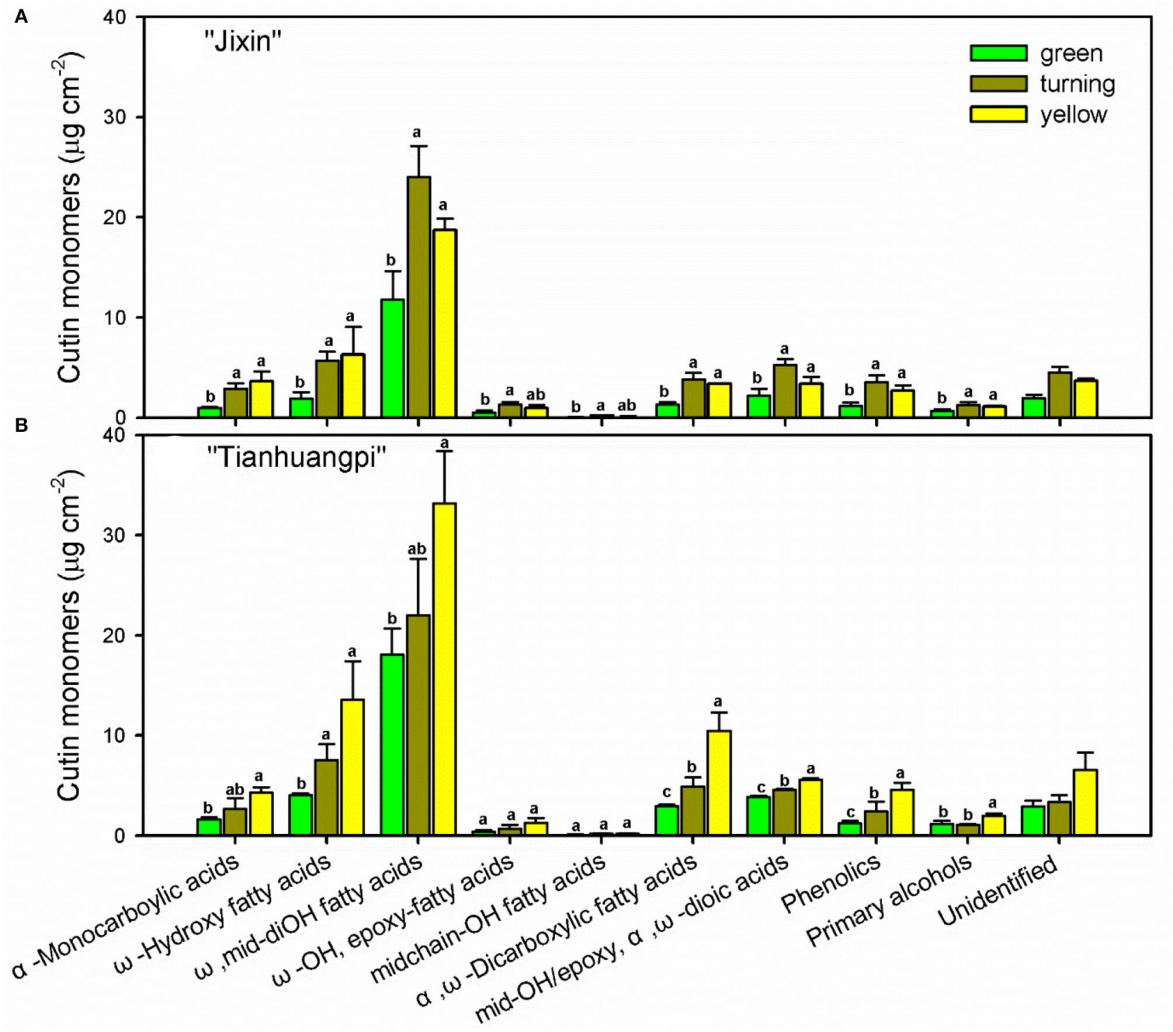
Chemical compositions of cutin monomers in wampee fruit (*Clausena lansium* (Lour.) Skeels). Fruits at the green, turning, and yellow stages of two cultivars **(A)** “Jixin” and **(B)** “Tianhuangpi” were comparatively analyzed. Data are given as means ± standard deviation (*n* = 5). Different letters indicate the significant differences at the level of 0.05.

Numerous cutin monomer families were detected in the wampee fruit cuticle. Similar to the changes in total cutin monomers, most classes of cutin monomers exhibited lower coverage in “Jixin” compared with “Tianhuangpi” (Figure 3). The homologs of ω-, mid-dihydroxy fatty acids (varying from 42.7 to 52.2% of total monomers) were the prominent cutin monomers, followed by α,ω-dicarboxylic acids with or without added hydroxy or epoxy groups, α-monocarboxylic acids with or without ω- or mid-chain hydroxy groups, primary alcohols, and phenolics (Figure 3). The most abundant monomer was 9(10),16-dihydroxyhexadecanoic acid, which ranged from 11.3 μg cm^−2^ (green) to 22.7 μg cm^−2^ (turning) in “Jixin” and between 17.4 μg cm^−2^ (green) and 31.8 μg cm^−2^ (turning) in “Tianhuangpi” (Supplementary Materials—cutin monomers). In addition, ω-OH C_16_ fatty acids (4.4 μg cm^−2^ to 5.0 μg cm^−2^ in “Jixin” and 7.7 μg cm^−2^ to 8.2 μg cm^−2^ in “Tianhuangpi”), α,ω-dicarboxylic C_16_ acids (5.1 μg cm^−2^ to 6.4 μg cm^−2^ in “Jixin” and 6.8 μg cm^−2^ to 9.4 μg cm^−2^ in “Tianhuangpi”), and 7(8)-OH-α,ω-dicarboxylic C_16_ acids (7.4 μg cm^−2^ to 9.8 μg cm^−2^ in “Jixin” and 6.9 μg cm^−2^ to 10.4 μg cm^−2^ in “Tianhuangpi”) were also detected as abundant monomers in wampee fruit cuticles (Supplementary Materials—cutin monomers). The other monomers were detected in <5 μg cm^−2^ and increased slightly during development (Figure 3 and Supplementary Materials—cutin monomers). Similarly, α-monocarboxylic acids with mid-OH (C_15_) or with ω-OH and mid-epoxy groups (C_18_) were detected in a small amount. Monofunctional fatty acids ranged from C_16_ to C_32_ with C_16_, C_18_, and C_30_ being the most abundant. Small amounts of primary alcohols, from C_24_ to C_31_, and coumaric acid and their derivatives as phenolics were also detected (Figure 3 and Supplementary Materials—cutin monomers). The most prominent components in cutin, C_16_- and C_18_-type monomers, exhibited a decreasing trend in their ratio from 9.1 to 6.5 in “Jixin” and from 10.8 to 7.4 in “Tianhuangpi” (Table 1).

### Chemical Composition of Cuticular Waxes

The accumulation of VLC aliphatic components in waxes varied from 1.9 μg cm^−2^ (green) to 4.2 μg cm^−2^ (turning) in “Jixin” and increased by 4-fold from 2.0 μg cm^−2^ (green) to 8.3 μg cm^−2^ (yellow) in “Tianhuangpi” (Table 1). The content of cyclics increased from 2.6 μg cm^−2^ (green) to 4.5 μg cm^−2^ (turning) in “Jixin” and from 4.1 μg cm^−2^ (green) to 5.4 μg cm^−2^ (yellow) in “Tianhuangpi” (Table 1). It is also noteworthy that the relative contents of VLC aliphatics and cyclics in total waxes appeared in stable ratios in “Jixin” (between 0.7 and 1.1) while increasing by 3-fold (0.5 to 1.5) in “Tianhuangpi” during fruit development (Table 1).

**Figure 4:**
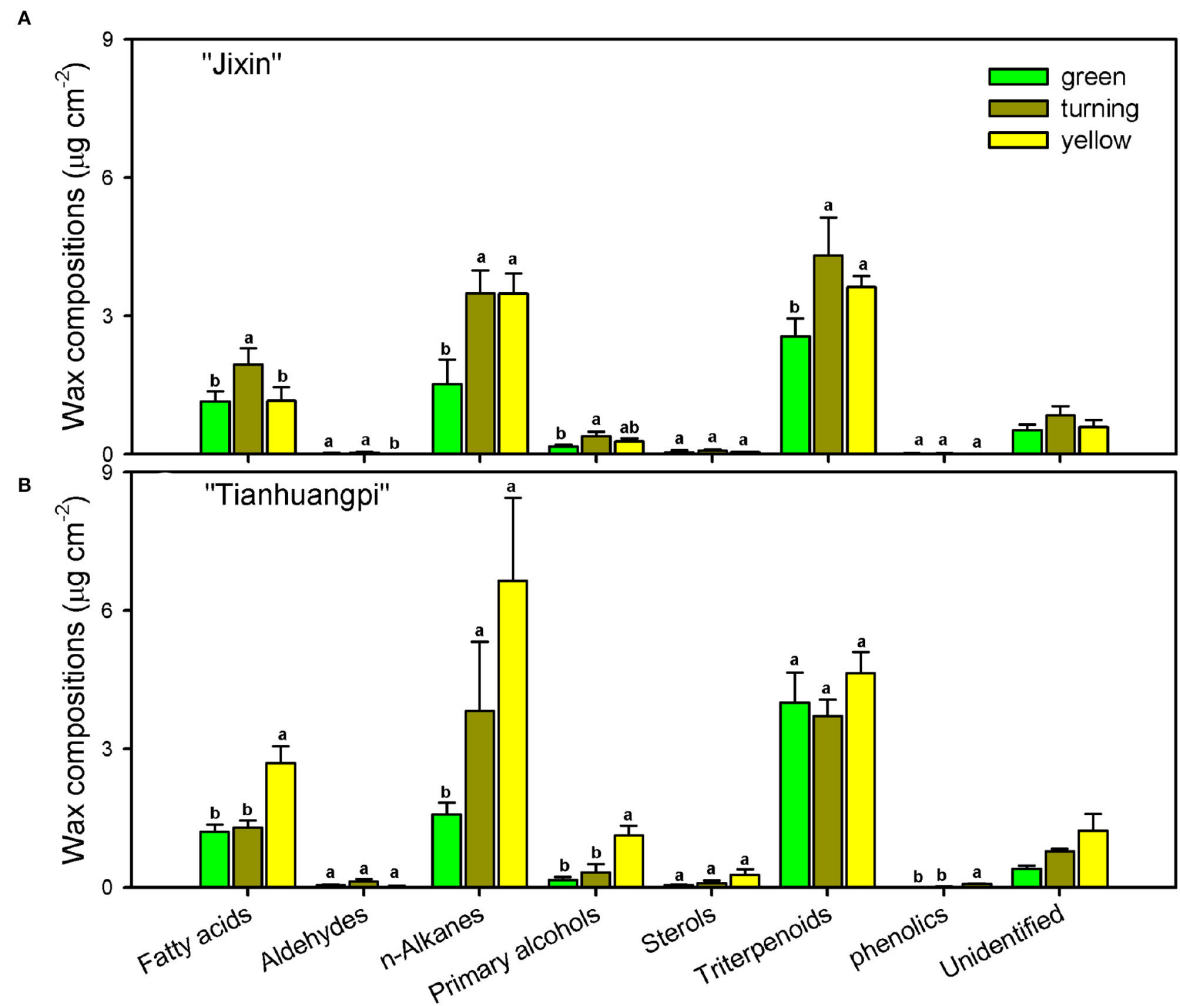
Chemical compositions of cuticular waxes in wampee fruit (*Clausena lansium* (Lour.) Skeels). Fruits at the green, turning, and yellow stages of two cultivars **(A)** “Jixin” and **(B)** “Tianhuangpi” were comparatively analyzed. Data are given as means ± standard deviation (*n* = 5). Different letters indicate the significant differences at the level of 0.05.

**Figure 5:**
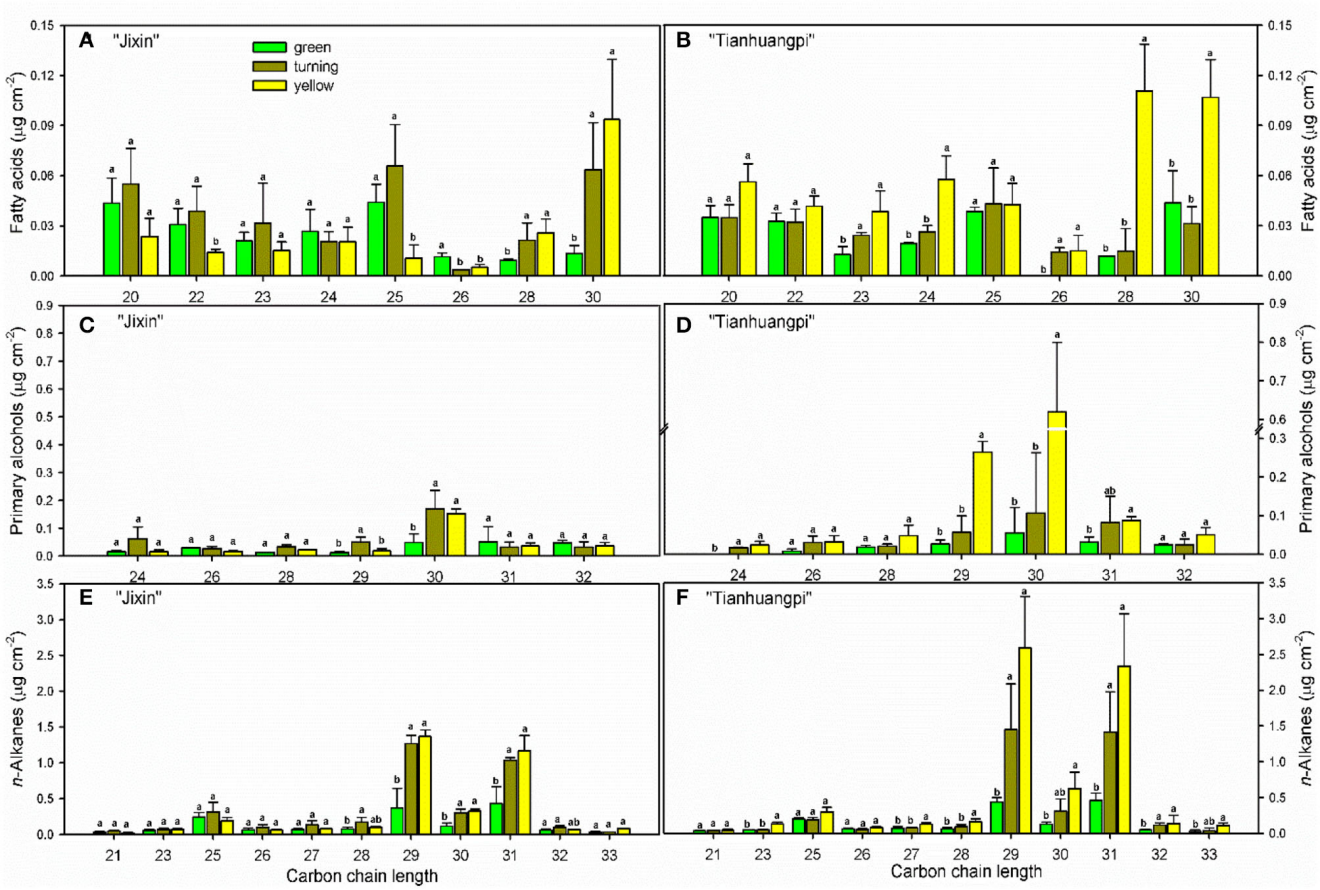
Carbon chain-length distribution and content of aliphatics in wampee fruit (*Clausena lansium* (Lour.) Skeels). **(A,B)** fatty acids, **(C,D)** primary alcohols, and **(E,F)** *n*-alkanes in fruits at the green, turning, and yellow stages of “Jixin” and “Tianhuangpi” were comparatively analyzed, respectively. Data are given as means ± standard deviation (*n* = 5). Different letters indicate the significant differences at the level of 0.05.

The aliphatic components were dominated by *n*-alkanes, followed by fatty acids, primary alcohols, and aldehydes in waxes (Figure 4 and Supplementary Materials—waxes). The most abundant VLC *n*-alkanes accumulated with homolog carbon chains from C_21_ to C_33_ and were dominated by C_29_ (6.0–14.9% in “Jixin”, and 5.9–15.3% in “Tianhuangpi”) and C_31_ (7.0–12.7% in “Jixin”, and 6.2–13.7% in “Tianhuangpi”), which were lower in the green stage than in other stages (Figure 5A and Supplementary Materials—waxes). Fatty acids from C_20_ to C_30_ were mainly even-numbered carbon chains with an increasing trend for C_28_ and C_30_ during development (Figure 5B and Supplementary Materials—waxes). Similar to fatty acids, primary alcohols accumulated as even-numbered carbon chains between C_24_ and C_32_ and changed mostly in C_30_ (Figure 5C and Supplementary Materials—waxes). Only a small amount of C_30_ for aldehydes was detected in wampee fruit waxes (Supplementary Materials—waxes).

**Figure 6:**
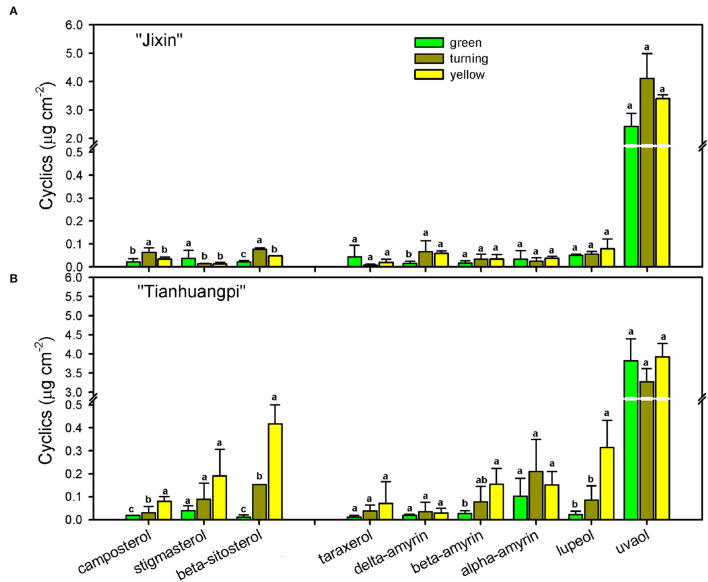
Changes in sterols and triterpenoids as cyclic pattern in wax mixtures of wampee fruit (*Clausena lansium* (Lour.) Skeels). Fruits at the green, turning, and yellow stages of two cultivars **(A)** “Jixin” and **(B)** “Tianhuangpi” were comparatively analyzed. Data are given as means ± standard deviation (*n* = 5). Different letters indicate the significant differences at the level of 0.05.

The cyclics in the waxes of wampee fruits were found to be mainly triterpenoids and sterols. Triterpenoids as the prominent cyclic components comprised various members, the most abundant being uvaol (37.2– 40.3% in “Jixin” and 23.9–51.2% in “Tianhuangpi”), followed by lupeol, α-amyrin, β-amyrin, σ-amyrin, and taraxerol (Figure 6). These triterpenoids were detected to maintain relatively stable levels from the green to yellow ripe stages in both “Jixin” and “Tianhuangpi” (Figure 6 and Supplementary Materials—waxes). Campesterol, stigmasterol, and β-sitosterol were detected as the sterol constituents, in which stigmasterol and β-sitosterol increased notoriously during development. In addition, small amounts of tocopherols as phenolic components were also detected (Figure 6 and Supplementary Materials—waxes).

### Cuticular Transpiration of Wampee Fruit

**Figure 7:**
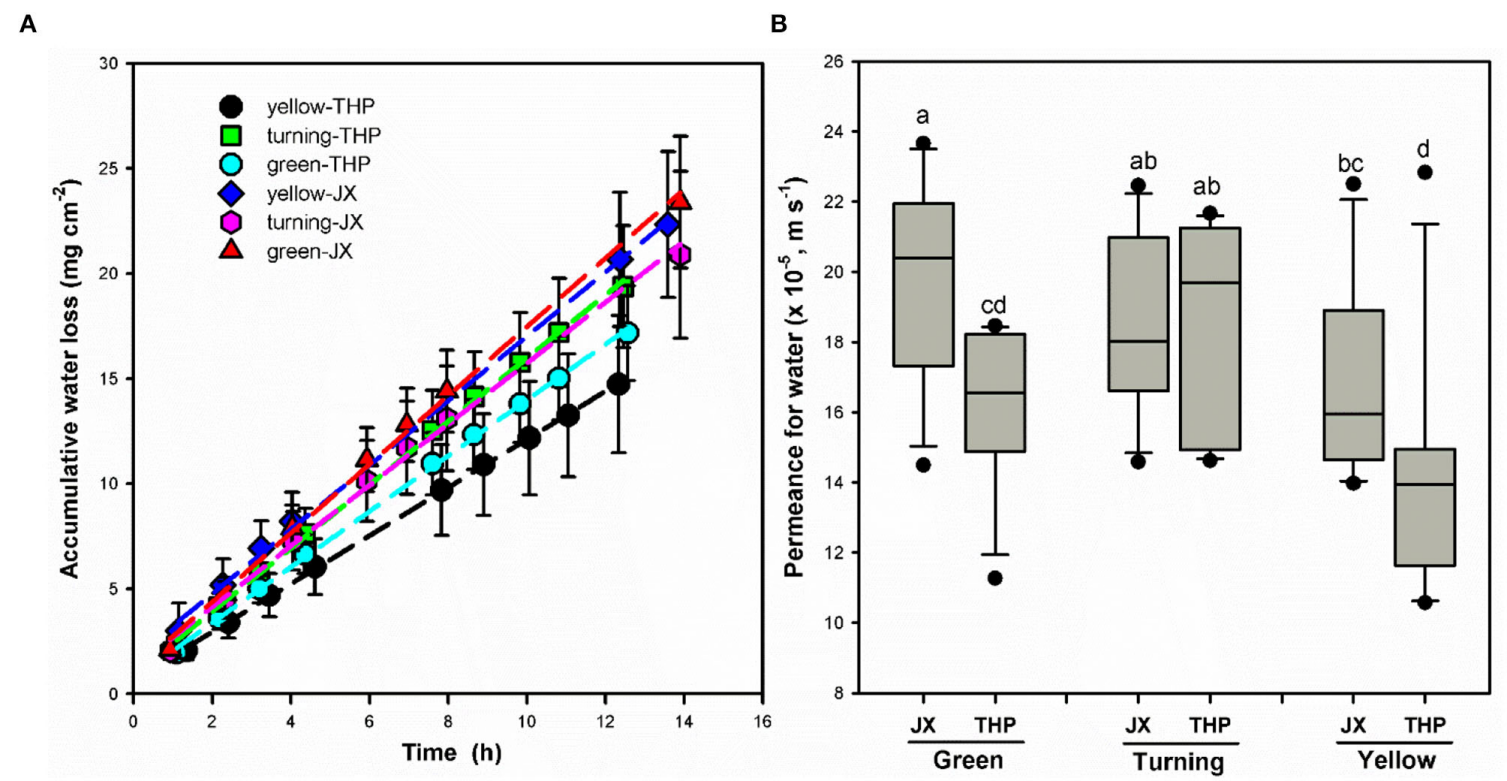
**(A,B)** Transpiration in wampee fruit (*Clausena lansium* (Lour.) Skeels) at the green, turning, and yellow stages of “Jixin” —JX and “Tianhuangpi” —THP. Data are given as means ± standard deviation (*n* = 15). Different letters indicate the significant differences at the level of 0.05.

Transpiration was measured at different developmental stages by continually recording the weight loss. As shown in Figure 7A, water loss in weight per unit of the surface area of wampee fruit at different stages exhibited linear changes over time (*r*^2^ > 0.99). Plant transpiration has been reported to be regulated either by the surface stomata exhibiting a dynamic process named drying curve or by the hydrophobic barrier contributed by the cuticle with linear changes of water loss (Burghardt and Riederer, 2003; Zeisler-Diehl et al., 2017). The transpiration of wampee fruit exhibited linear changes during the measured time period extending up to 24 h or even longer. This indicated a single main factor affecting the barrier property for transpiration in wampee fruit. Taking the driving force into account, permeance for water in wampee fruit was lower in “Tianhuangpi” than in “Jixin” at both green and yellow stages. In contrast, water permeability exhibited the lowest levels at the yellow ripe stage in both “Jixin” and “Tianhuangpi” (Figure 7B). Permeance for water varied between 2.0 × 10^−4^ m s^−1^ (green) and 1.7 × 10^−4^ m s^−1^ (yellow) in “Jixin” and from 1.6 × 10^−4^ m s^−1^ (green) to 1.4 × 10^−4^ m s^−1^ (yellow) in “Tianhuangpi” (Figure 7B).

## Discussion

Wampee fruit is rich in phytochemicals with antioxidant and anticancer activities (Prasad et al., 2009; Ye et al., 2019). However, the main part of peel tissues, the outer cuticle, has not been previously studied. The cutin matrix was constructed by numerous monomers dominated by 9/10,16-dihydroxy fatty acids, varying from 38.9% in yellow “Tianhuangpi” to 50.3% in green “Jixin” of total monomers (Figure 3 and Supplementary Materials—cutin monomers). Monomers of ω-, mid-dihydroxy fatty acids as the main cutin components have been reported in many other fruits, especially in tomatoes (Leide et al., 2007), peppers (Parsons et al., 2013), pitayas (Huang and Jiang, 2019), olives (Huang et al., 2017), and selected northern berries (Järvinen et al., 2010). However, the main monomer components in the wampee fruit cuticle were different from those of the citrus fruit (species in the same family as wampee), in which ω-hydroxy fatty acids with mid-chain oxo-group dominated in cutin monomers (Wang et al., 2016). Besides the cutin monomers, *n*-alkanes ranged from 21.4% in green “Tianhuangpi” to 39.3% in yellow “Tianhuangpi” as the main aliphatics, while triterpenoids varied between 28.2% in yellow “Tianhuangpi” and 53.6% in green “Tianhuangpi” as cyclic components (Figure 5A and Supplementary Materials—waxes). The *n*-alkanes and triterpenoids have also been found as prominent compounds in waxes in tomatoes (Leide et al., 2007) and cherries (Belge et al., 2014).

The cutin monomers and waxes accumulated in similar chemical classes but exhibited cultivar- and development-related variability in wampee fruit. The coverage of waxes and cutin monomers, as well as their variety of components, was overall lower in “Jixin” than in “Tianhuangpi” (Figures 3, 4 and Supplementary Materials—cutin monomers and waxes). Similar cultivar-related differences were reported in sweet cherries (Belge et al., 2014), olives (Diarte et al., 2019), grape berries (Yang et al., 2021), and northern berries (Järvinen et al., 2010). In addition, cuticular components in mass per unit of fruit surface area were accumulated in lower levels in the green fruit and were highest at the turning stage in “Jixin”, while they increased gradually in “Tianhuangpi” during development (Figures 3, 4 and Supplementary Materials). The surface waxes increased gradually in mass in bayberry and grape berry fruits and peaked at 85 and 81 DAA, respectively, while exhibiting a slightly decreasing trend thereafter (Simpson and Ohlrogge, 2016; Arand et al., 2021). It has also been mentioned that both cutin monomers and waxes exhibited an increasing trend during development in tomato (Leide et al., 2007) and cherry (Peschel et al., 2007). In citrus fruits, the distant relatives of wampee, the accumulation of cutin monomers increased with the expansion of the fruits and peaked at 180 DAA (color turning stage), whereas waxes increased through the whole developmental period till 240 DAA (Wang et al., 2016). As a result, the cultivar and developmental time differences in wampee fruit exhibited the typical berry characteristics on the basis of the changes in cuticular chemical composition.

The cuticle functions as an extracellular membrane covering plant surfaces and is largely affected by the chemical constituents and their structural arrangements (Riederer and Schreiber, 2001). On the basis of the above chemical analysis, wampee fruit forms a similar berry fruit cuticle, which plays an important role against nonstomatal transpiration, pathogen infection, and fruit cracking. The *n*-alkanes, dominated by C_29_ and C_31_, as well as the main carbon chain of C_30_ for fatty acids, primary alcohols, and aldehydes were the main VLC aliphatic components (Figure 5 and Supplementary Materials—waxes). In addition, the VLC aliphatics in mass per surface area were lower at the green stages and increased following the development of the fruit in both “Jixin” and “Tianhuangpi.” Consequently, the overall carbon chain aliphatics indicated by average chain length shifted from C_28_ to C_29_ during development (Table 1). As the efficiency of the hydrophobic barrier to transpiration is largely contributed by the VLC aliphatic compounds (Leide et al., 2007), changes in VLC components may influence the water status in wampee fruit.

Similar to the changes in cuticular chemicals, transpiration of wampee fruit also exhibited cultivar and developmental time differences. It has been reported that transpiration of plant tissues was mainly regulated by stomata and the outermost hydrophobic cuticle. When stomata are open, the dynamic water loss is evidenced as a drying curve, which shows a rapid initial water loss and slows down following the closing of stomata (Burghardt and Riederer, 2003). In contrast, the behavior is linear for transpiration *via* nonstomatal surfaces or when most stomata are closed (Zeisler-Diehl et al., 2017). In this study, the linear dynamic changes of water loss even during prolonged measurements, indicating a single barrier for transpiration, were largely contributed by the cuticle in wampee fruit. Water permeability was generally lower in “Tianhuangpi” than in “Jixin” and showed the highest level at the green stage with a decreasing trend during development (Figure 7). Simultaneously, the overall VLC aliphatics on total waxes, especially the major pattern of *n*-alkanes, C_29_ and C_31_, increased in both mass per surface area and relative level during fruit development (Supplementary Materials—waxes). Accordingly, a negative correlation between the accumulation of VLC *n*-alkanes and the water permeability was found (*r*^2^= 0.84, *p* < 0.01). It has been verified that the VLC aliphatics, especially hydrocarbons, are pivotal in regulating the transpiration in plants to adapt to water-deficit stresses (Kosma et al., 2009; Patwari et al., 2019; Dimopoulos et al., 2020). The increase of VLC aliphatics, in both amount and relative level, may enhance the hydrophobic crystalline zones in the cuticle (Riederer and Schreiber, 1995; Huang et al., 2017), thus slowing down the transpiration in wampee fruit during development.

It is noteworthy that triterpenoids, another major family of wax compounds, accumulated steadily in mass per surface area but decreased in the relative level during fruit development (Figures 5, 6). It has been mentioned that the triterpenoids were largely detected in the intracuticular layer in plants (Jetter and Riederer, 2015; Arand et al., 2021). In wampee fruit, uvaol as the major triterpenoid was released together with cutin monomers, which was embedded in the cutin matrix. The abundant triterpenoids were implicated to function as fillers embedding in the cutin matrix to plasticize and enhance the mechanical properties of the cuticle (Tsubaki et al., 2013; España et al., 2014). Cutin monomers polymerize as a matrix to provide scaffolding for the accumulation of waxes and also enhance the mechanical support in the cuticle (Fich et al., 2016). In tomatoes, cutin deficit was found to accelerate fruit softening and susceptibility to pathogen infection (Isaacson et al., 2009; Yeats et al., 2012). During the development of wampee fruit, the amount of triterpenoids and cutin monomers increased per surface area, while the relative level of total wax content and total cutin monomers decreased remarkably (Figures 3, 6 and Supplementary Materials). These changes may induce the alteration in the arrangement of cuticular chemicals on a spatial level.

In addition, berry fruits are susceptible to cracking during development or at the ripening stage such as in cherries, grape berries, plums, and tomatoes (Khadivi-Khub, 2014). As wampee fruit exhibits similar characteristics to these berry fruits, the increase of chemical components in the cuticle might be important to enhance the barrier so as to protect from fruit cracking. On the one hand, the fruit exhibits vigorous metabolism and high transpiration during development. Thus, it needs to accumulate waxes and cutin to enhance mechanical properties maintaining the fruit integrity during the expansion of fruit (Knoche and Lang, 2017). Though lower amounts of cuticular components accumulated in green fruit, the higher relative contents of triterpenoids and cutin monomers were important to strengthen the mechanical properties of the cuticle to protect fruit integrity. On the other hand, with the expansion of the fruit, the relative content of cuticular chemicals decreased in cyclics and cutin monomers, which may form a less tight cuticle, inducing cracking in ripe berries compared with young fruits (Knoche et al., 2004). In addition, as compared to green fruits, water permeability was lower in ripe fruit, which might be because of the maintenance of fruit quality following the ripening of the fruit (Figure 7). It should be noted that *n*-alkanes as one of the main wax components exhibited an increasing trend for both mass per surface area and the relative content level (Figure 5 and Supplementary Materials—waxes). It has also been proposed that the accumulation of *n*-alkanes was probably taking part in enhancing the tolerance to cracking in cherry fruits (Ríos et al., 2015). Therefore, the decline of the relative level in cyclics and cutin monomers may broaden the space in the cuticle to embed the *n*-alkanes, thus forming a better barrier to transpiration in wampee fruit at the ripe stage.

In conclusion, this study reports the detailed chemical composition of waxes and cutin monomers as well as their potential effects on the transpiration barrier properties in wampee fruit. The cuticular chemical composition and transpiration properties exhibited cultivar and developmental time differences following the expansion of wampee fruit. Cuticular waxes were dominated by *n*-alkanes and triterpenoids, while ω-, mid-chain dihydroxy fatty acids were the prominent cutin monomers. The contents of cuticular chemical composition were higher in “Tianhuangpi” than in “Jixin” and were lower in green fruits. Transpiration was higher in the green fruit and decreased during development. In addition, the increase of *n*-alkanes, dominated by C_29_ and C_31_, in both mass per area and relative content levels provided further insights into the importance of hydrocarbons to form hydrophobic barriers in the cuticle. To the best of our knowledge, this study provides the first approach to cuticle chemicals as well as to the physiological functions, taking transpiration as an example in wampee fruit. Further study on the effect of cuticles on fruit cracking and microbial infection is necessary.

## Data Availability Statement

The original contributions presented in the study are included in the article/Supplementary Material, further inquiries can be directed to the corresponding authors.

## Author Contributions

HH designed and performed most of the experiments and prepared the draft of the manuscript. LW and DQ contributed to part of the experiments and data analyses. YL took part in revising the manuscript and participated in funding acquisition. All authors approved the final version of the manuscript.

## Funding

This work was supported by the Funding for Excellent Young Scientists of Guangdong Academy of Agricultural Sciences (R2021YJ-YB1005), Wild Plant Resources (Fruit Trees) Survey, Collection and Evaluation Project (2021KJ269), Funding of Young Scientist Cultivation for Institute of Fruit Tree Research, Guangdong Academy of Agricultural Sciences (2021-107), Common Technical Innovation Team of Guangdong Province on Preservation and Logistics of Agricultural Products (2022KJ145), the Innovation Team of Modern Agricultural Industry Technology System in Guangdong Province of China (2021KJ116), the Special Financial Fund of Foshan-Guangdong Agricultural Science and Technology Demonstration City Project (2021), the National Tropical Plants Germplasm Resources Center, the Guangdong Basic and Applied Basic Research Foundation (2019A1515110611), Funding from Guangxi Key Laboratory of Fruits and Vegetables Storage-Processing Technology (2021-01), the “Pearl River Talent Plan” for Young Scientists Program of Guangdong Province [2018(2)], and special fund for scientific innovation strategy-construction of high level Academy of Agriculture science of Guangdong Academy of Agricultural Sciences (R2019QD-012).

## Conflict of Interest

The authors declare that the research was conducted in the absence of any commercial or financial relationships that could be construed as a potential conflict of interest.

## Publisher's Note

All claims expressed in this article are solely those of the authors and do not necessarily represent those of their affiliated organizations, or those of the publisher, the editors and the reviewers. Any product that may be evaluated in this article, or claim that may be made by its manufacturer, is not guaranteed or endorsed by the publisher.
